# Programmable flip-metasurface with dynamically tunable reflection and broadband undistorted transmission

**DOI:** 10.1515/nanoph-2023-0635

**Published:** 2024-01-09

**Authors:** Cong Wang, Xiangteng Li, Hongchen Chu, Baiyang Liu, Shenhao Miao, Ruwen Peng, Mu Wang, Yun Lai

**Affiliations:** National Laboratory of Solid State Microstructures, School of Physics, and Collaborative Innovation Center of Advanced Microstructures, Nanjing University, Nanjing 210093, China; Department of Electronics and Electrical Engineering, Southern University of Science and Technology, Shenzhen, 518055, China

**Keywords:** programmable metasurface, tunable reflection, undistorted transmission

## Abstract

We introduce a programmable flip-metasurface that can dynamically control the reflection while leaving the transmitted wavefront undistorted in an ultra-broad spectrum, i.e., the same as that of the incidence. This metasurface is constructed by unique meta-atoms that can be dynamically switched between two flip states, which correspond to the spatial inversion of each other. Due to the reciprocity principle and spatial inversion symmetry, the transmission is independent of the flip states, regardless of the frequency. While the reflection can be conveniently controlled by tuning the flip states. Dynamical steering of the reflected waves, such as diffuse reflection, focusing, and beam-splitting, is numerically and experimentally validated along with unaffected transmission. Our finding opens an approach to dynamically modulate reflections without affecting transmission, which could have broad potential applications ranging from wireless communications to stealth technology.

## 1 Introduction

Metasurfaces [1], [2], [3], [4] consist of an array of planar meta-atoms with spatially varying structures and, hence, distinct responses when interacting with the incident electromagnetic waves. Waves scattered from the metasurface can then be flexibly manipulated as needed by precisely arranging the meta-atoms to impose desired distributions of phase, amplitude, and polarization on the incidence. Up to now, plenty of novel mechanisms in metasurfaces have been revealed, including generalized reflection/refraction law [5], [6], propagating-to-evanescent wave conversion [7], [8], [9], high-efficiency Huygens’ metasurfaces [10], [11], polarization conversion [12], [13], [14], Pancharatnam–Berry-phase metasurfaces [15], [16], reciprocity-protected metasurfaces [17], [18], [19], [20], and so on. These findings further inspire novel phenomena and devices based on metasurfaces, such as achromatic metalenses [21], [22], [23], [24], orbital-angular-momentum beam generation [25], [26], high-efficiency and high-capacity holograms [27], [28], [29], [30], and ultrathin invisibility cloaks [31]–[35], etc. By introducing tuning mechanisms, including optical, magnetic, thermal, and electric tunings in metasurfaces, the response of each single meta-atom and then the functionality of the whole metasurface become dynamically tunable [36]–[44].

Based on tunable metasurfaces, the notion of programmable coding metasurfaces (PCM) was introduced as a digital counterpart to traditional metasurfaces, aiming to exert precise control over electromagnetic (EM) waves [45]. Most typical PCMs operate in either reflection or transmission modes [45], [46], [47], [48]. One of the most crucial requirements on PCM designs for wireless communication applications is full-space wave steering, i.e., simultaneously manipulating the reflection and transmission waves. One standard method to realize full-space PCMs is to restrict distinct incident waves with different frequencies, polarizations, or incident angles separately to the reflection and transmission half spaces [49], [50], [51]. However, this method relies on two distinguished channels, hindering its practical applications. Recently, a study has demonstrated that it is possible to achieve simultaneously independent control over transmission and reflection within a shared polarization and frequency channel [52]. But, the bandwidth is limited, and the spatial sampling of the phase shift pattern has a lower density compared to conventional metasurfaces.

**Figure 1:**
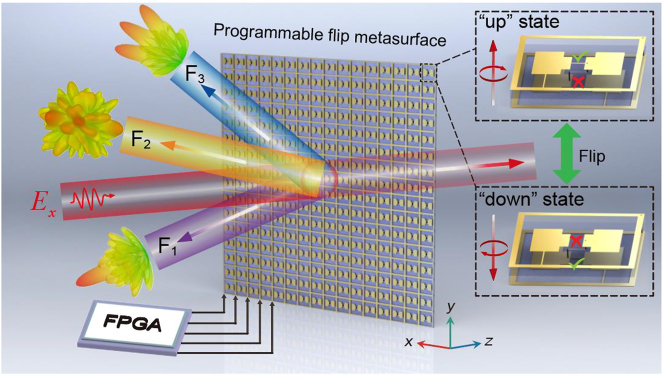
Conceptual illustration of the programmable flip-metasurface. The reconfigurable multi-functionality (*F*_1_–*F*_3_) of reflection and undistorted transmission wavefront are simultaneously achieved through controlling the up and down states of meta-atoms of the programmable flip-metasurface.

In this study, we demonstrate a programmable flip-metasurface (PFM) that is capable of dynamically manipulating reflection while keeping the transmitted wavefront the same as that of the incidence in an ultra-broad spectrum, as schematically shown in Figure 1. The PFM is composed of an array of meta-atoms with two-metal-layer structure and a positive-intrinsic-negative (PIN) diode on each layer as shown in inset of Figure 1. Each meta-atom supports two different states, where the PIN on either the top layer or the bottom layer is on. Analogous to the two spin states of electrons, such two states of meta-atoms are denoted as “up” state and “down” state. The switch between these two states is equivalent to physically flipping the meta-atom, i.e., applying spatial inversion to it. Such a switch can be realized by controlling the on/off states of PIN diodes in the meta-atoms. According to the mechanism of flip-metasurface [17], [19], [53], such two states of a meta-atom have the same transmission phase but distinct reflection phases. Therefore, real-time control of the reflection wavefront can be realized through modulation of the states of each meta-atom by employing a field-programmable-gate-array (FPGA)-based system to control the PIN diodes therein. Meanwhile, the wavefront in the transmission is unaffected no matter what the arrangement of the states of meta-atoms is. To verify the performance of the PFMs, we present a dynamic switchover between multi-functionalities of reflection, including the focusing, beam splitting, and diffuse reflection, and simultaneously unchanged transmission. Both numerical simulation and microwave measurement have validated the functionality of the PFMs. Our work paves the road towards dynamic and high-spatial-resolution manipulations of reflection without affecting transmission, which may yield many inspiring applications in fields like wireless communications and stealth technologies.

## 2 Results

**Figure 2:**
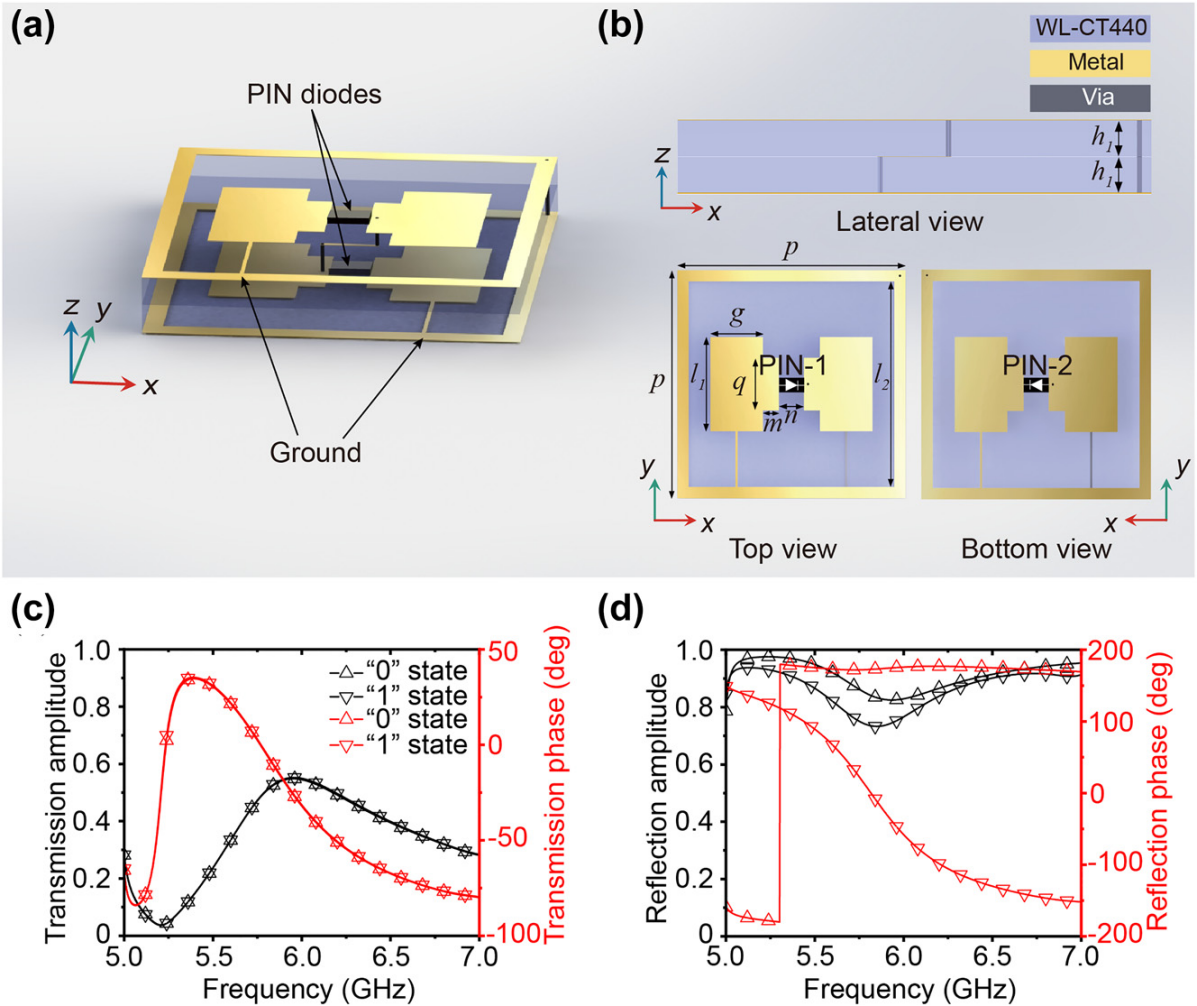
Meta-atom designed for a PFM. (a, b) Perspective view (a), lateral view, top view, and bottom view (b) of the meta-atom with geometric parameters of *p* = 20 mm, *l*_1_ = 8.25 mm, *l*_2_ = 18 mm, *g* = 4.6 mm, *q* = 4.6 mm, *m* = 1.4 mm, *n* = 2.2 mm, *h*_1_ = 1.5 mm. (c) The calculated transmission amplitudes and phases of the two states of the meta-atom. (d) The calculated reflection amplitudes and phases of the two states of the meta-atom. The upward triangle symbols represent the “0” state mete-atom, and the downward triangle symbols represent the “1” state meta-atom.

As a practical example of PFM, we design a component meta-atom constructed by two identical layers containing a PIN diode (Skyworks SMP1320-079LF) connecting two metal patches, as illustrated in Figure 2(a) and (b). The two layers are separated by a dielectric spacer with a dielectric constant of 4.38 and a loss tangent of 0.004. The bias network located between the two layers is not shown here. A diagonal pair of passive patches, i.e., the left patch on the top layer and the right patch on the bottom layers, are wired to the ground square frame. Another diagonal pair of active patches, i.e., the right patch on the top layer and the left patch on the bottom layers, is connected through metallic holes. The geometric parameters of the meta-atom are specified in the figure caption of Figure 2. The passive patches are connected to a reference voltage of 1.8 V. The active patches are connected to either a high bias voltage of 0 V or a low bias voltage of 3.3 V through the middle biasing lines (not shown here), resulting in two different states, i.e., “top diode on and bottom diode off” and “top diode off and bottom diode on,” which correspond to the “up” state and “down” state respectively. The equivalent circuits of a PIN diode can be considered as an RLC series connection with parameters *L* = 0.5 nH, *C* = 0.24 pF when the PIN diode is in the off states, whereas with parameters *R* = 0.5 Ω, *L* = 0.7 nH when the PIN diode in the on states.

Simulations are carried out by using the finite-element method. The simulated transmission and reflection coefficients of both the “up” state and “down” state of the meta-atom under the normally incident *x*-polarized incident wave are shown in Figure 2(c) and (d), respectively, where the upward triangle symbols depict the “up” state mete-atom and the downward triangle symbols depict the “down” state meta-atom. From Figure 2(c), it is seen that the transmission amplitude and phase of the meta-atom between the “up” and “down” states are the same. In contrast, Figure 2(d) illustrates that the two states have distinct reflection phases and almost identical reflection amplitudes. The reflection phase difference reaches the maximum value of 180° at 5.836 GHz. From the perspective of coding metasurface, meta-atoms supporting such two states with 180° phase difference are typical 1 bit coding elements. Therefore, the “up” and “down” states are also referred to as “0” and “1” states in the following.

**Figure 3:**
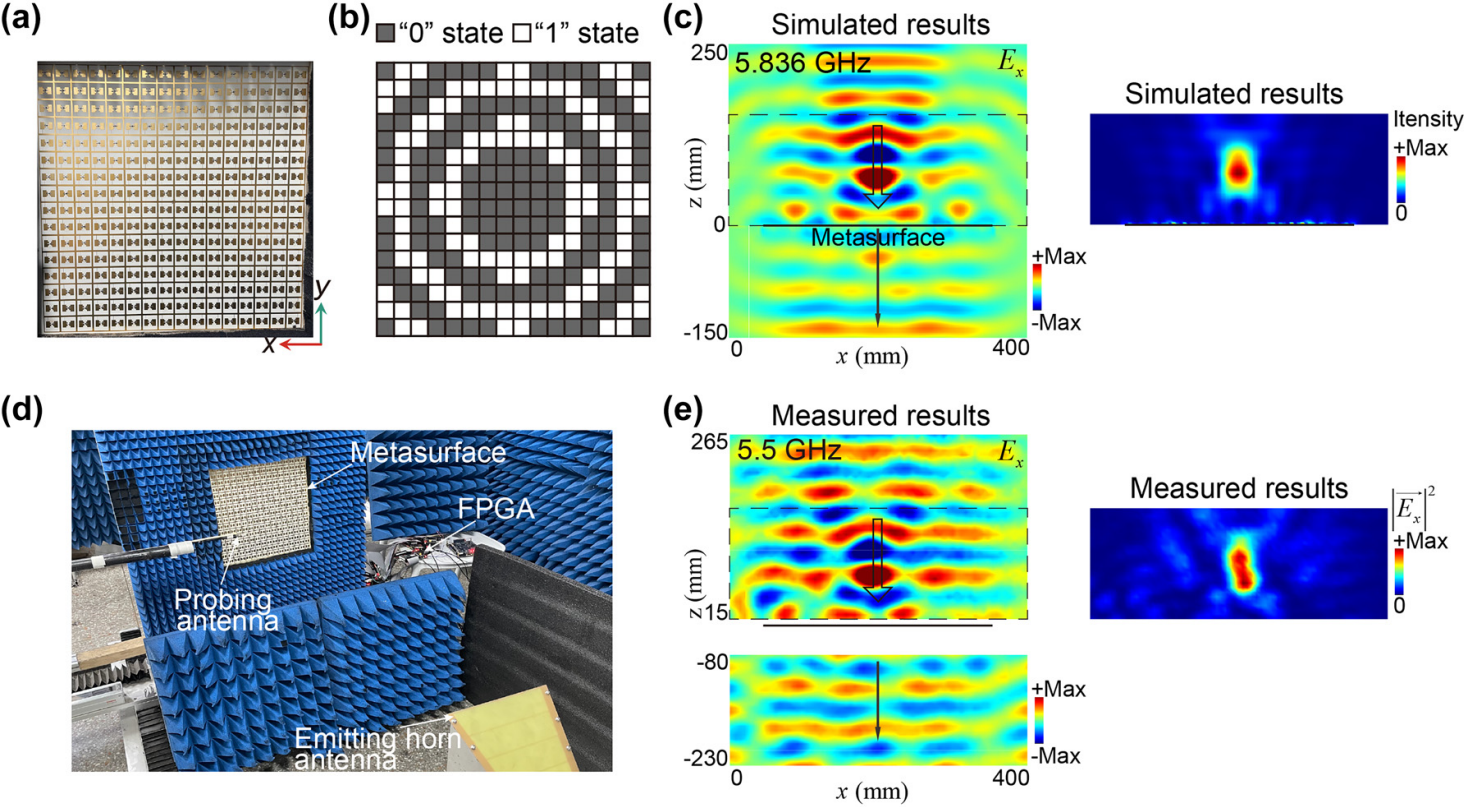
The focusing functionality (*F*_1_) in the reflection and simultaneously undistorted transmission wavefront enabled by a PFM. (a) Photograph of the fabricated metasurface. (b) Designed state pattern of the PFM under an *x*-polarized incident Gaussian wave propagating along the −*z* direction at 5.836 GHz. (c) The simulated *E*_*x*_ component of the scattered field distributions on the *x* − *z* plane (left panel). The simulated field-intensity enhancement in the reflection space (right panel). (d) Experiment setup for near-field scanning. (e) The measured *E*_*x*_ component of the scattered fields of the PFM at 5.5 GHz (left panel). The measured field-intensity distribution ( ${\left|\vec{E_{x}}\right|}^{2}$ ) of the reflected fields in reflection at 5.5 GHz (right panel). Due to the considerable thickness of the absorber around the PFM, some areas near the PFM can’t be measured.

We then design a PFM consisting of 16 × 16 meta-atoms with a total size of 320 mm × 320 mm. Such a PFM is fabricated by the commercial standard printed circuit board (PCB) technology. The fabricated PFM is shown in Figure 3(a). As the first illustrative example, the Fresnel wave zone plate coding pattern is designed for the focusing functionality (*F*_1_) when an *x*-polarized incident Gaussian wave propagates along the −*z* direction. In a circular binary phase Fresnel zone plate, the boundaries of each zone can be expressed as:

(1) $$r_{m}=\sqrt{mf_{d}\lambda +{\left(m\lambda /2\right)}^{2}},\;\;\;\;\;m=1,2,3\cdot \cdot \cdot$$

where *f*_*d*_ is the focal length and *λ* is the wavelength of the working frequency in free space. According to equation (1), we have designed a circular binary phase Fresnel zone plate to focus the incident wave with a focal length *f*_*d*_ of 60 mm at 5.836 GHz, the corresponding phase coding pattern is shown in Figure 3(b). We first employ full wave simulations to validate the functionality of this PFM. The simulated *E*_*x*_ component of the scattered field distributions on the *x* − *z* plane at 5.836 GHz is shown in the left panel in Figure 3(c), indicating the focusing effect in the reflection space and that the transmitted wave possesses a flat wavefront and its propagation direction is the same as that of the incident Gaussian beam. We note that in principle such an undistorted transmission wavefront applies to frequencies ranging from DC to frequency where the diffraction effect starts to occur. Because the identical transmission amplitudes and phases of the meta-atoms working in up and down states are protected by reciprocity and are frequency-independent. However, for a practical metasurface, on the one hand, at low frequencies where the wavelength is much larger than the size of meta-atoms, the reflected and transmitted properties of the meta-atom can be barely tailored by engineering its geometric parameters. Therefore, here we consider a finite frequency range of 4–8 GHz. The simulated transmitted wave of the PFMs at frequencies of 4 GHz, 6 GHz, and 8 GHz are separately shown in the Supplementary Materials, where it is found that the wavefronts of transmitted waves through PFM maintain the planar wavefront of incidence, demonstrating the ultra-broadband property of the undistorted transmission. We then calculate the field-intensity enhancement in the *x* − *z* plane at 5.836 GHz. The enhancement is 10.85 times at the focal point, and the focal length is 68.85 mm, as can be found from the simulated field-intensity distribution of the reflection shown in the right panel in Figure 3(c). The field enhancement at the focal point is considerable in the frequencies ranging from 5.4 GHz (6.21 times) to 6.1 GHz (5.22 times). Specifically, at 5.7 GHz, this enhancement reaches a remarkable factor of 11.30, which indicates that the PFM has a notable focusing effect with a bandwidth of around 0.7 GHz.

The near-field scanning experimental setup within a microwave anechoic chamber is depicted in Figure 3(d). To generate a quasi-plane-wave incidence, an emitting horn antenna was employed. A probing antenna was used for electric field scanning on the horizontal plane (*x* − *z* plane). These two antennas were connected to two ports of a network analyzer (KEYSIGHT N5224B) to obtain both the magnitude and phase of the electric field at the position of the probing antenna. The measured *E*_*x*_ component of the scattered fields of the PFM, exhibiting the focusing functionality (*F*_1_) at 5.5 GHz, is depicted in Figure 3(e). The top and bottom panels display the reflected and transmitted fields, respectively. Additionally, the measured field-intensity distribution ( ${\left|\vec{E_{x}}\right|}^{2}$ ) of the reflected fields at 5.5 GHz is plotted in the right panel of Figure 3(e). The measured results in Figure 3(e) reveal a clear focal spot with high field intensity near the focal point and a nearly plane-wave transmission wavefront. The measured results closely align with the simulation outcomes, affirming the capabilities of the PFM to achieve both focusing effects in reflection and an undistorted transmission wavefront. The frequency discrepancy between measurements and simulations should be attributed to several reasons. Firstly, in the sample fabrication, additional metal wires and irregularly shaped solder joints are introduced on the outer sides of the diode’s pins to attain a good connection, both of which are not considered in simulations, where ideal metal wires are used to connect the diode. Secondly, in the experiments, the incident wave generated by a horn antenna has a convex wavefront. While the simulations employed a perfect Gaussian beam with a specific beam waist and focus distance. Thirdly, in simulations, we model the PIN diode as an equivalent RLC series circuit, which is different from the true response of a practical PIN diode in the fabricated sample.

**Figure 4:**
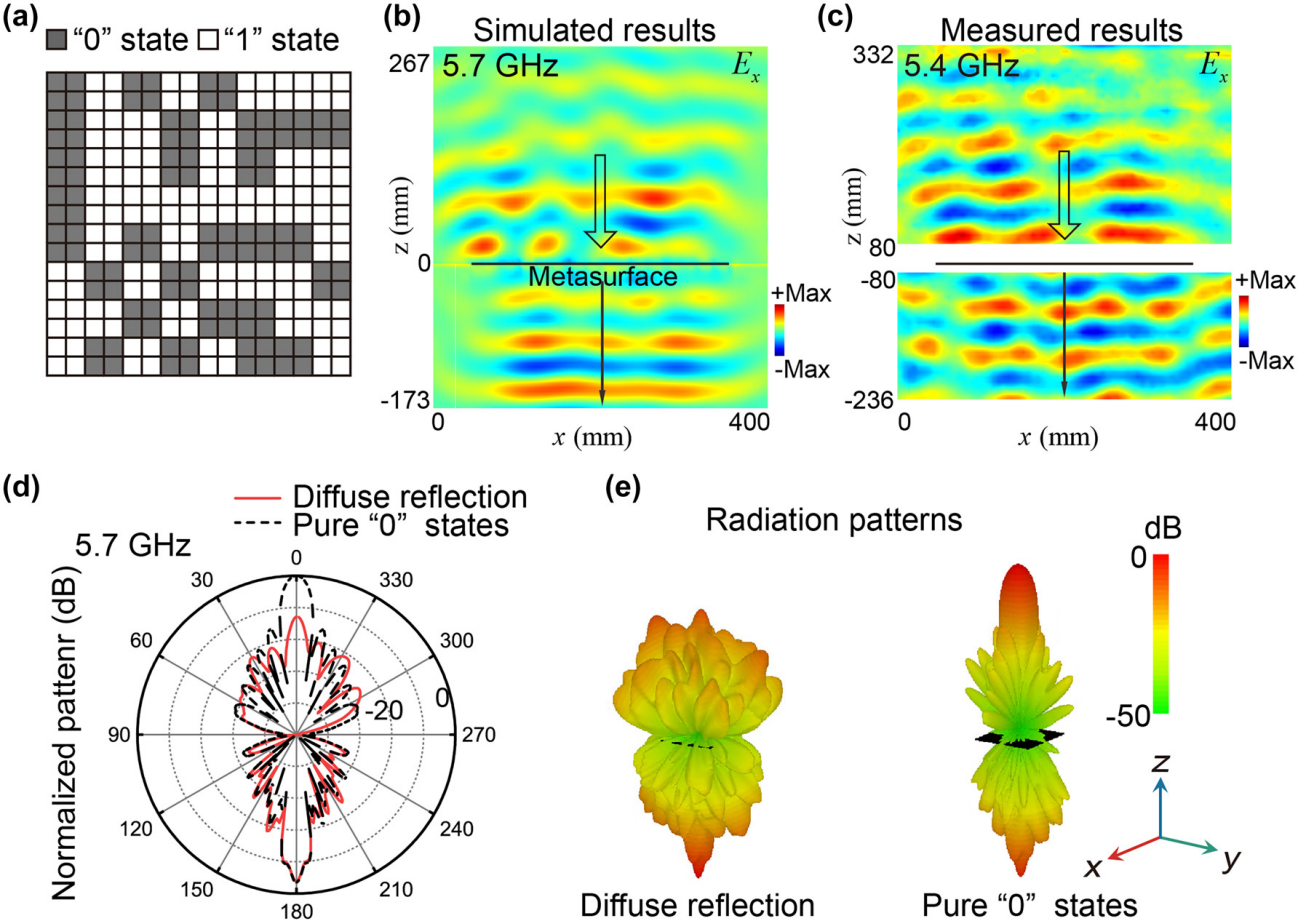
The diffuse reflection functionality (*F*_2_) and simultaneously undistorted transmission wavefront enabled by a PFM. (a) Designed state pattern of the PFM under an *x*-polarized incident Gaussian wave propagating along the −*z* direction at 5.836 GHz. (b) The simulated *E*_*x*_ component of the scattered field distribution on the *x* − *z* plane. (c) The measured *E*_*x*_ component of the scattered fields of the PFM at 5.4 GHz. (d) The simulated 2D far-field radiation patterns of the PFM (solid lines) and the reference pure “0” state metasurface (dashed lines). (e) The simulated 3D far-field radiation patterns of the PFM (left panel) and the reference pure “0” state metasurface (right panel).

As the second illustrative example, we tune this PFM to possess a random state distribution so that it can generate diffuse reflection (*F*_2_) and an undistorted transmission wavefront. A supercell of square 2 × 2 “0” state meta-atoms or “1” state meta-atoms is utilized as the metasurface block and referred to as the 1 bit coding block. The PFM is composed of 8 × 8 coding blocks. The desired random pattern configuration of the “0” state coding blocks and “1” state coding blocks can be optimized by using the artificial bee colony algorithm. The optimized random pattern is shown in Figure 4(a). The scattered field distributions of the designed PFM are simulated under the illumination of an *x*-polarized incident Gaussian wave propagating along the −*z* direction. Figure 4(b) shows the simulated *E*_*x*_ component of the scattered field distributions on the *x* − *z* plane at 5.7 GHz. It is seen that the transmitted wave maintains the plane wavefront, while the wavefront of the reflected wave is unordered. The simulated transmitted wave of the PFMs at frequencies of 4 GHz, 6 GHz, and 8 GHz are separately shown in the Supplementary Materials, exhibiting ultra-broadband undistorted transmissions. From the measured scattered electric fields shown in Figure 4(c), we can also observe a diffuse reflection and a quasi-plane-wave transmission at 5.4 GHz. To quantitatively study the diffusing ability of the PFM, we also simulated the far-field radiation patterns of the PFM. For comparison, a PFM with only “0” state meta-atoms is also investigated. As illustrated in Figure 4(d) and (e), the 2D and 3D radiation patterns of the PFM at 5.7 GHz exhibit the optimal suppression of the specular reflection, which exceeds 12.86 dB, when compared to the reference pure “0” state metasurface. The deviation between 5.7 GHz and the frequency where the reflection phase difference is largest, i.e., 5.836 GHz, should be attributed to that both the differences in reflection phase and amplitude affect the diffusion performance of the PFM. However, the transmission lobes in both the random PFM and pure “0” state metasurface are nearly identical in both directions and intensities. Compared to the reference metasurface, the suppression of the specular reflection is more than 10 dB within the frequency range from 5.5 GHz to 5.75 GHz, indicating a bandwidth of around 0.25 GHz. The simulated and measured results verify the capabilities of the PFM to achieve both diffuse reflection and an undistorted transmission wavefront.

**Figure 5:**
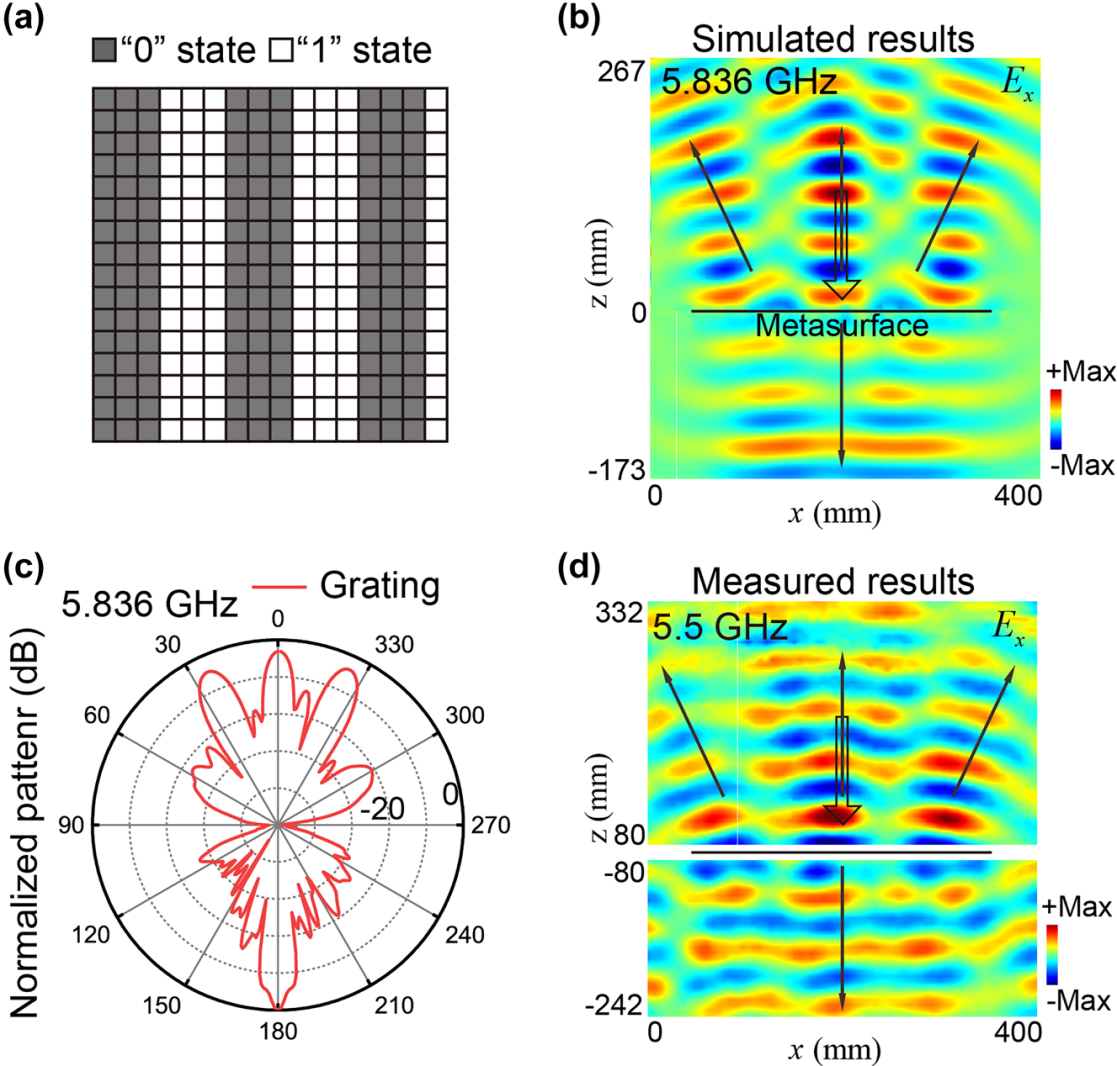
The beam splitting functionality (*F*_3_) in the reflection and simultaneously undistorted transmission wavefront enabled by a PFM. (a) Designed state pattern of the PFM under *x*-polarized incident Gaussian wave propagating along the −*z* direction at 5.836 GHz. (b) The simulated *E*_*x*_ component of the scattered field distribution on the *x* − *z* plane. (c) The simulated 2D far-field radiation patterns at 5.836 GHz. (d) The measured *E*_*x*_ component of the scattered fields of the PFM at 5.5 GHz.

As the last example, the reflected functionality of the PFM is set as beam splitting (*F*_3_). The grating-like state pattern of the PFM is illustrated in Figure 5(a). In this case, a zero-order and two first-order diffracted beams in the reflection are allowed. The deflection angle for these two first-order diffracted beams can be theoretically predicted using the grating theory, which can be expressed as follows:

(2) $$\theta_{m}=\sin^{-1}\left(m\lambda /T\right),$$

where *T* is the period of the coding pattern, and *m* is the order of diffraction. Under the *x*-polarized incident Gaussian wave propagating along the −*z* direction at 5.836 GHz, the PFM will deflect the normally incident wave to directions with angles of 0° and ±25.3° in the *x* − *z* plane according to Equation (2). The simulated *E*_*x*_ component of the scattered field distributions on the *x* − *z* plane at 5.836 GHz is shown in Figure 5(b). The corresponding 2D far-field radiation pattern is presented in Figure 5(c). These simulation results reveal that the reflected wave is split into three beams with deflection angles of *θ* = 0° and *θ* = ±25°, respectively, and that the transmitted wave keeps the planar wave front of the incidence. The simulated transmitted wave of the PFMs at frequencies of 4 GHz, 6 GHz, and 8 GHz are separately shown in the Supplementary Materials, exhibiting ultra-broadband undistorted transmissions. These results align well with the theoretical predictions. We note that for phase-type grating composed of elements possessing strictly out-of-phase reflection with identical amplitude, all even diffraction orders should vanish [54]. The origin of the zero-order reflection of the PFM here should be attributed to the distinct reflection amplitude of the two states stemming from the effect of the bias network, which breaks the identity between meta-atoms. The measured *E*_*x*_ component of the scattered fields of the PFM, demonstrating the beam splitting functionality (*F*_3_) at 5.5 GHz, is illustrated in Figure 5(d). The measured results align well with the simulation results, validating the capabilities of the PFM to achieve both beam splitting in the reflection and an undistorted transmission wavefront.

## 3 Discussion and conclusion

The proposed PFM can be easily extended to full-space PCMs [52], [55]–[59] by stacking a transmission-type PCM [47] on the back of the PFM, which has been intensively studied. In this configuration, the reflection is manipulated directly by the PFM, and the undistorted transmission through the PFM is further tailored by the PCM to the desired wavefront. Compared to previous full-space PCM, the whole region of the metasurface contributed to forming both the reflection and transmission wavefront.

It is worth noting that the manipulated reflections are usually valid in a limited band due to the dispersive reflection phase difference between the two states. Several common mechanisms might be introduced to broaden the operating frequency range in reflection, such as dispersion compensation [60] and interference effect [53].

The proposed PFM can manipulate the reflection in real-time without affecting the transmission with the same polarization, which is beyond most of the previous full-space metasurfaces limited to tailoring two orthogonal polarization channels. A polarization-independent PFM can be compatible with a broader range of application scenarios. One of the most traditional methods to extend the current PFM design to the polarization-independent configuration is to adopt *C*_4v_ symmetric metallic structures in the meta-atom design [19], [61]. In this circumstance, each meta-atom contains more PIN diodes. Therefore, the complexity of the bias network might be significantly increased. Optimizing the topology and geometry of the meta-atoms might help to release some requirements on the bias network.

In summary, we have proposed and experimentally verified a novel PFM that can dynamically manipulate the reflection while maintaining the transmission wavefront undistorted. The PFM consists of an array of meta-atoms constructed by two identical metal layers with a controllable PIN diode. Such a meta-atom can be switched between two states, which are flip counterparts to each other, by an FPGA control system. The reciprocity principle and space-inversion operation guarantee the unaffected transmission when the phase coding pattern of the metasurface in reflection is changed. Both the simulation and measured results have affirmed the outstanding performance of the PFM. The demonstrated PFM provides a flexible platform in multifunctional and full-space electromagnetic manipulations, which could achieve practical applications in the field of wireless communication and scattering manipulation, especially in cases where transmission wavefront is required to be undistorted, such as radomes.

## Supplementary Material

Supplementary Material Details
