# Supplementary material for: Programmable flip-metasurface with dynamically tunable reflection and broadband undistorted transmission
Source: Nanophotonics. 2024 Jan 9;13(12):2151–9. doi: 10.1515/nanoph-2023-0635 (PMC11501368; doi:10.1515/nanoph-2023-0635)
Supplement: Supplementary file 1 — Supplementary Material Details [file j_nanoph-2023-0635_suppl_001.docx]

Supplementary Information

**Programmable flip-metasurface with dynamically tunable reflection and broadband undistorted transmission**

Cong Wang, Xiangteng Li, Hongchen Chu*, Baiyang Liu, Shenhao Miao, Ruwen Peng*, Mu Wang* and Yun Lai*

**^*^Corresponding author:** **Hongchen Chu, Ruwen Peng, Mu Wang and Yun Lai,** National Laboratory of Solid State Microstructures, School of Physics, and Collaborative Innovation Center of Advanced Microstructures, Nanjing University, Nanjing 210093, China; E-mail: chuhongchen@nju.edu.cn (H. Chu), rwpeng@nju.edu.cn (R. Peng), muwang@nju.edu.cn (M. Wang), laiyun@nju.edu.cn (Y. Lai). https://orcid.org/0000-0002-8577-5870 (H. Chu). https://orcid.org/0000-0003-0424-2771 (R. Peng). https://orcid.org/0000-0002-3823-1272 (M. Wang). https://orcid.org/0000-0002-0040-9274 (Y. Lai).

1. **The broadband undistorted transmission of the PFM**

Figs. S1(a-c) show the simulated transmitted wave of the PFMs operating in F1, F2, and F3 at the frequencies of 4 GHz, 6 GHz, and 8 GHz respectively. For comparison, when the PFM is removed, the incident wave at the same region is shown in Figs. S1(d). From Fig. S1, it is found that the wavefronts of transmitted waves through PFM with different functionalities maintain the planar wavefront of incidence in a broad frequency range, demonstrating the ultra-broadband property of the undistorted transmission.


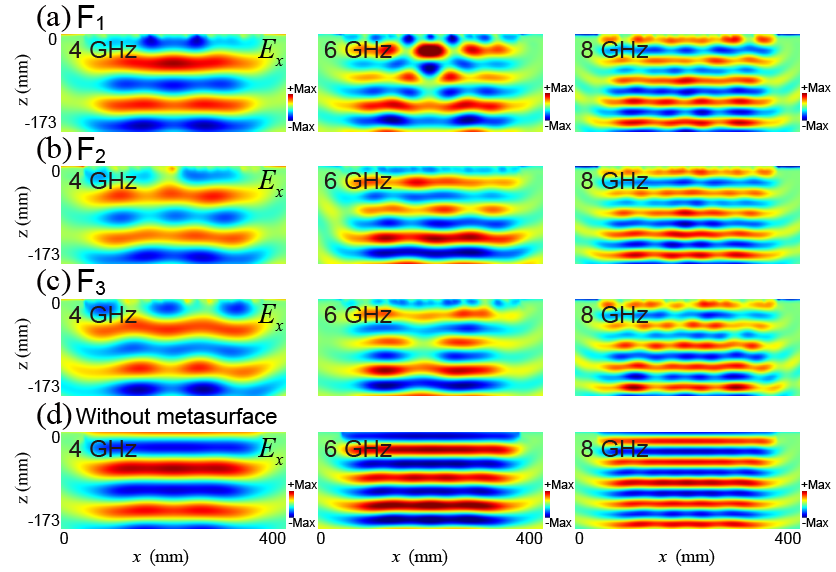


Fig. S1. The simulated transmitted field distribution (*E_x_*) of the PFM under an *x*-polarized incident Gaussian wave propagating along the -z direction on the *x*-*z* plane at 4GHz, 6GHz, and 8GHz. The PFM exhibits F1(a), F2(b), and F3(c) respectively. (d) The simulated transmitted field distribution (*E_x_*) when the PFM is removed.
